# Comparison of three assembly strategies for a heterozygous seedless grapevine genome assembly

**DOI:** 10.1186/s12864-018-4434-2

**Published:** 2018-01-17

**Authors:** Sagar Patel, Zhixiu Lu, Xiaozhu Jin, Padmapriya Swaminathan, Erliang Zeng, Anne Y. Fennell

**Affiliations:** 1 Agronomy, Horticulture and Plant Science Department and BioSNTR, 247 McFadden BioStress Laboratory, South Dakota State University, Brookings, SD 57006 USA; 2 Department of Computer Science, University of South Dakota, Vermillion, SD USA; 3 Department of Biology, University of South Dakota, Vermillion, SD USA

**Keywords:** De novo genome assembly, Heterozygous, *Vitis vinifera*, Seedless grape, Sultanina, PLATANUS, ALLPATHS-LG, METASSEMBLER, GapCloser

## Abstract

**Background:**

De novo heterozygous assembly is an ongoing challenge requiring improved assembly approaches. In this study, three strategies were used to develop de novo *Vitis vinifera* ‘Sultanina’ genome assemblies for comparison with the inbred *V. vinifera* (PN40024 12X.v2) reference genome and a published Sultanina ALLPATHS-LG assembly (AP). The strategies were: 1) a default PLATANUS assembly (PLAT_d) for direct comparison with AP assembly, 2) an iterative merging strategy using METASSEMBLER to combine PLAT_d and AP assemblies (MERGE) and 3) PLATANUS parameter modifications plus GapCloser (PLAT*_GC).

**Results:**

The three new assemblies were greater in size than the AP assembly. PLAT*_GC had the greatest number of scaffolds aligning with a minimum of 95% identity and ≥1000 bp alignment length to *V. vinifera* (PN40024 12X.v2) reference genome. SNP analysis also identified additional high quality SNPs. A greater number of sequence reads mapped back with zero-mismatch to the PLAT_d, MERGE, and PLAT*_GC (>94%) than was found in the AP assembly (87%) indicating a greater fidelity to the original sequence data in the new assemblies than in AP assembly. A de novo gene prediction conducted using seedless RNA-seq data predicted > 30,000 coding sequences for the three new de novo assemblies, with the greatest number (30,544) in PLAT*_GC and only 26,515 for the AP assembly. Transcription factor analysis indicated good family coverage, but some genes found in the VCOST.v3 annotation were not identified in any of the de novo assemblies, particularly some from  the MYB and ERF families.

**Conclusions:**

The PLAT_d and PLAT*_GC had a greater number of synteny blocks with the *V. vinifera* (PN40024 12X.v2) reference genome than AP or MERGE. PLAT*_GC provided the most contiguous assembly with only 1.2% scaffold N, in contrast to AP (10.7% N), PLAT_d (6.6% N) and Merge (6.4% N). A PLAT*_GC pseudo-chromosome assembly with chromosome alignment to the reference genome *V. vinifera*, (PN40024 12X.v2) provides new information for use in seedless grape genetic mapping studies. An annotated de novo gene prediction for the PLAT*_GC assembly, aligned with VitisNet pathways provides new seedless grapevine specific transcriptomic resource that has excellent fidelity with the seedless short read sequence data.

**Electronic supplementary material:**

The online version of this article (10.1186/s12864-018-4434-2) contains supplementary material, which is available to authorized users.

## Background

The grapevine genus *Vitis* contains more than 50 species which are distributed in Asia, Europe and North and Central America [1]. In spite of the importance of this fruit crop and ongoing breeding efforts in table, raisin and wine grapes, there are very few genome sequences available. The first grapevine genome assembly, and fourth higher plant genome assembled, was developed for *Vitis vinifera* (PN40024), a highly homozygous inbred genotype, using Sanger technology in 2007 [2]. A heterozygous *V. vinifera* Pinot Noir genome and Cabernet Sauvignon genome were assembled using long read technology, 454 sequencing and PacBio respectively [3, 4]. It will be very beneficial to have multiple genomes available, as this aids in SNP detection with improved statistical power/lower false-positive rate and genetic coverage from genotype by sequencing approaches for improved map development and genetic analyses [5, 6]. Short read assemblies could fill this gap with the higher accuracy and lower cost, however, development of grapevine assemblies presents a challenge as grapevine cultivars are highly heterozygous and have strong inbreeding depression [2, 3, 6–10]. Recent advances in assembler algorithms provide the potential to utilize the less expensive high-throughput short read technology for developing high quality de novo assemblies enhancing the ability to identify novel genes, structural variants and SNP cataloging for genomic studies and marker assisted breeding [6, 8, 11]. A short read genome assembly of *V. vinifera* ‘Sultanina’, the main source of seedlessness for table grape breeding, was developed using ALLPATHS-LG, presenting important sequence resources to the community [12]. Genome assembling, however, is an evolving process and it is valuable to compare different assembly strategies and improve genome assemblies using new algorithms and continuously emerging RNA-seq data. Here we developed three de novo assemblies using the public ‘Sultanina’ genome sequence datasets [12] and de novo gene predictions using seedless RNA-seq data [13]. The quality of each assembly was evaluated relative to the recently updated *V. vinifera* reference genome (PN40024 12X.v2) [14] and a ‘Sultanina’ ALLPATHS-LG assembly (AP) [12]. PLATANUS software [11], a De Bruijn graph based assembler, developed to efficiently assemble short read sequences while maintaining heterozygosity, was used to develop a default de novo PLATANUS assembly (PLAT_d). In addition, two methods of scaffold size and continuity increase were employed: 1) METASSEMBLER [15] was used iteratively to develop a merged assembly (MERGE) from the PLAT_d and AP assemblies; 2) PLATANUS with parameter modifications [11] and two gap closing cycles using GapCloser (PLAT*_GC) [16]. The assembly strategies allow comparison between the two software in default mode and the two strategies for increasing scaffold size and continuity. In addition, seedless Thompson/Sultanina grapevine RNA-seq [13] datasets were used to perform a de novo gene prediction for all four assemblies, the predicted coding sequences were fully annotated and compared with *V. vinifera* (PN40024 12X.v2, VCOST.v3 annotation) gene models [14]. The comparisons among assembly strategies gained the following insights 1) Assembler in default mode (AP and PLAT_d) which informs users with RAM limitation on assembler differences; 2) Development of a hybrid assembly combining PLAT_d and AP that is compared with PLATANUS gap-closing; 3) The final PLAT*_GC de novo assembly, gene prediction, and pseudo-chromosomes provide an improved assembly and valuable resources for the grapevine scientific community.

## Methods

The public ‘Sultanina’ sequence data (accession #SRP26420) [17] used in the AP assembly [12] was assembled using PLATANUS assembler [11]. *V. vinifera* ‘Sultanina’ DNA sequence reads (186G bases in 1577 million reads) were downloaded from NCBI [17] for developing new PLATANUS related assemblies. The existing genome assembly and “novel” genes sequences of *V. vinifera* ‘Sultanina’ were downloaded from http://vitisdb.cmm.uchile.cl/publicationmaterial/ [12]. The *V. vinifera* (PN40024 12X.v2 and VCOST.v3 annotation) reference genome was downloaded from URGI database [18] and BAC sequences were downloaded from CRIBI [19]. The EST dataset of *V. vinifera* deposited in NCBI was downloaded (on 02/05/2016). Public RNA-seq data of seedless grape (BioProject accession #275778) [13] were downloaded and used for gene prediction in AP, PLAT_d, MERGE, and PLAT*_GC assemblies.

### De novo heterozygous genome assembly of *V. vinifera* L. ‘Sultanina’

Before genome assembly, duplicate Illumina reads were removed by FastUniq [20]. Reads were corrected using Quake [21] with the following parameters: minimum length of reads ≥ 70 bp and minimum quality ≥ 20. The filtered reads were used to identify heterozygosity using JELLYFISH [22] with –m 19 option and with GenomeScope [23]. A de novo genome assembly was developed using PLATANUS (version 1.2.4) [11] with default parameters (PLAT_d). The previously published ALLPATHS-LG assembly (AP) and PLAT_d were merged iteratively using METASSEMBLER [15]. The PLAT_d was used as the primary assembly and the AP assembly was used as the secondary assembly during merging process. An in-house python script was designed to execute METASSEMBLER [15] iteratively until no improvements in scaffold size for the merged assembly could be obtained. The resultant merged assembly is referred to as “MERGE”. A third assembly (PLAT*_GC) was developed using PLATANUS [11] with parameter modification followed by GapCloser [16]: 1) In the first ‘assemble’ step we changed two parameters from default (−u 0.1 and –d 0.3) to –u 0.2 and -d 0.3 with the other parameters remaining at default. The ‘-u’ parameter determines the maximum difference for bubble crush, the larger value increases the number of bubbles to be merged and if the heterozygosity in the species is high then large values should be used. The ‘-d’ parameter is for maximum difference for branch cutting and a smaller value increases the accuracy. These parameters were used to increase the number of heterozygous contigs remaining after the assemble step. 2) In the scaffold step, three parameters were changed from default (−s 32, −v 32, and –u 0.1) to -s 20, −v 20, and -u 0.2 with the remaining parameters at default. The ‘-s’ parameter is for the mapping seed length and ‘-v’ is the minimum overlap length and the ‘-u’ parameter is the same as described in above ‘assemble’ step. If the adjacent contigs have overlap (length > = 20) and are properly close to each other, the contigs are joined. 3) In the gap closing step the default parameters (−s 32, −vo 32, −vd 32, −ed 0.05) were changed to -s 20, −vo 20, −vd 20 and −ed 0.1. The ‘-vo’ parameter is for minimum overlap length among each read in OLC gap closing and ‘-vd’ parameter is for minimum overlap length between contig and edge seq in De Bruijn gap closing. Smaller values of ‘-vo’ and ‘-vd’ increase the number of gaps to be closed. The ‘-ed’ parameter is for maximum error rate among gap edge seq in De Bruijn gap closing, a larger value increases the number of gaps closed. These assemblies were used with GapCloser [16] to get final PLAT*_GC assembly. All assemblies have been publically deposited (http://openprairie.sdstate.edu/vitis_vinifera_sultanina/1).

The new assemblies (PLAT_d and PLAT*_GC) were performed on Linux server equipped with Intel X86–64 processor, 32 cores with 1 TB RAM and took 3 days for each assembly. The MERGE assembly was obtained on Linux server with 3.0GHz 8-Core Intel Xeon E5 processor and 12GB RAM. Each merge iteration took approximate 5 days and after five iterations, the assembly converged.

### *V. vinifera* L. ‘Sultanina’ genome assembly evaluation

The statistics for the four assemblies (AP, PLAT_d, MERGE and PLAT*_GC) were obtained using Assemblathon script [24] and the 486,205,130 base pair (bp) size of the *V. vinifera* reference genome (PN40024 12X.v2) was used for genome size estimation value [18]. Total Assemblathon statistics are found in Additional file 1 (a, b, c). Cumulative assembly sizes were compared using QUAST [25] with *V. vinifera* (PN40024 12X.v2, VCOST.v3) reference genes and genome [18]. BUSCO [26] was used with the latest plant data sets (embryophyta odb9) in genome mode for all four assemblies to assess the completeness of the conserved proteins in the assemblies.

### Analysis of sequence fidelity maintenance by zero-mismatch mapping back of filtered reads

All filtered reads used for genome assembly were also used for zero-mismatch mapping back to all four assemblies using Bowtie2 [27]. The SAM files of Bowtie2 [27] mapping results were converted to BAM files using SAMtools [28], and then the alignment statistics were obtained using the flagstat option of SAMtools.

### Mapping the EST and BAC from *V. vinifera* to seedless assemblies

GMAP [29] was used with default parameters to map the EST sequences of *V. vinifera* from NCBI to the four de novo assemblies. MUMmer [30] package was used for comparison of *V. vinifera* BAC sequences [19] with the four assemblies: 1) BAC sequences were aligned to scaffolds of the four assemblies using nucmer with -mum option. 2) The output results from nucmer were filtered using delta-filter with the -g option. 3) The filtered results were considered for show-coords program and the coordinates of the resulting alignments were obtained. All four assemblies were aligned with *V. vinifera* (PN40024 12X.v2) reference genome by the same method as was used for the BAC sequences. The alignments that represented the longest length (top-hit) for each BAC and scaffolds of all four assemblies aligned with *V. vinifera* (PN40024 12X.v2) reference genome, were summed (top-hits-length).

### SNP calling of *V. vinifera* L. ‘Sultanina’

Repeat mapped reads were removed using rmdup in bowtie2 [27] results. The SNPs were called against reference genome *V. vinifera* (PN40024, 12X.v2) using the mpileup of SAMtools [28] with default parameters. These SNPs were filtered by VCFtools [28] using a window of 10, a minimum depth 8 and a minimum quality 40. SNP effect was predicted using SnpEff program [31].

### De novo gene prediction and functional characterization of the *V. vinifera* L. ‘Sultanina’ assemblies

RNA-seq data of Thompson/Sultanina seedless grape (BioProject accession #275778) was used for de novo gene prediction [13]. The RNA-seq data was first filtered using TRIMMOMATIC [32] (quality score ≥ 20 and read length ≥ 70), and then mapped separately to each of the four assemblies using bowtie2 [28] and tophat2 [33]. A custom repeat library was created for each of the four assemblies and pseudo-chromosome assembly of PLAT*_GC using RepeatScout [34] and then repeats were masked by RepeatMasker [35]. The masked genome assemblies were further considered for de novo gene prediction with BRAKER-1 [36] using the RNA-seq [13] data of seedless grape. Predicted coding sequences from the four assemblies were further characterized using Blast2GO [37]. First BLASTX was performed using the nr database and parameters: E value 1.0E-3; number of blast hits 1, word size 3, HSP length cutoff 33 and eukaryote selected as the taxonomy. The BLASTX results for each of the coding sequences were further searched for enzyme classification using: InterPro, GO (gene ontology) and KEGG pathway analysis by Blast2GO [37]. BLASTX and BLASTP of coding and protein sequences were conducted for all four assemblies against the latest (2017) reference proteins of *V. vinifera* (PN40024, 12X.v2, VCOST.v3) [14]. Default parameters and the non-hit sequences of BLASTX and BLASTP were then searched as described previously, using Blast2GO [37] with BLASTX and BLASTP. Predicted genes were then functionally annotated using the *V. vinifera* annotation and VitisNet pathways [7] providing seedless transcriptomic resources. Transcription factors were identified using PlantTFDB [38]. The RAV transcription factor subfamily of AP2/ERF TF family was examined using ClustalW in MEGA7 [39] to align AP, PLAT*_GC and VCOST.v3 and phylogenetic tree constructed by neighbor-joining using the pair-wise deletion option and 1000 bootstrapping permutations.

### Orthologous genes

All de novo predicted protein sequences for the four assemblies and the latest *V. vinifera* (PN40024 12X.v2 VCOST.v3) protein sequences [14] were considered for finding orthologous genes by OrthoMCL [40]. All protein sequences were filtered using ≥ minimum length of 10 amino acids. Then the OrthoMCL steps were performed as described in OrthoMCL manual [40]. The results from OrthoMCL [40] were visualized by OrthoVenn [41].

### Pseudo-chromosome development

All four assemblies were aligned with *V. vinifera* (PN40024 12X.v2, VCOST.v3) reference genome [14] by MUMmer [30] and then the same steps were performed as noted for the BAC alignment. The scaffolds that mapped with the longest alignment on *V. vinifera* chromosomes were placed in artificial chromosomes from 1 to 20. Duplicate mapped scaffolds were removed after this step, keeping the scaffold with the longest alignment with reference genome. The scaffolds that did not map with reference genome *V. vinifera* (PN40024 12X.v2) were grouped into a chromosome 21*.* Synteny blocks between the genome assemblies of *V. vinifera* ‘Sultanina’ (AP, PLAT_d, MERGE, and PLAT*_GC) and *V. vinifera* (PN40024 12X.v2) were computed by SyMAP [42]. SyMAP provides the number of anchors have alignment lengths >10 kb with the reference genome. All four assemblies were separately mapped with *V. vinifera* using the promer option of MUMmer [30] by SyMAP [42]. The assemblies were mapped reciprocally with the *V. vinifera* reference genome (i.e. AP, MERGE and MERGE, AP) so that visualization of blocks could be presented in same orientation for each assembly.

## Results

### *D*e novo heterozygous assembly of ‘Sultanina’

The three de novo assemblies are presented to provide 1) the most direct comparison of PLATANUS default (PLAT_d) with the published assembly AP; 2) different methods of closing gaps and improving continuity of assembly (MERGE and PLAT*_GC); 3) comparisons of PLAT*_GC as final assembly with AP. An average memory peak 850 Gb was observed for PLAT_d and PLAT*_GC assemblies in the ‘assemble’ step and an average 30 Gb memory peak was observed for ‘scaffold’ and ‘gap close’ steps in PLATANUS assembler. Iterative merging required 5 days for each merging round (5 rounds total) on a standard Linux server (3.0GHz 8-Core Intel Xeon E5 processor, 12GB RAM).

**Fig. 1:**
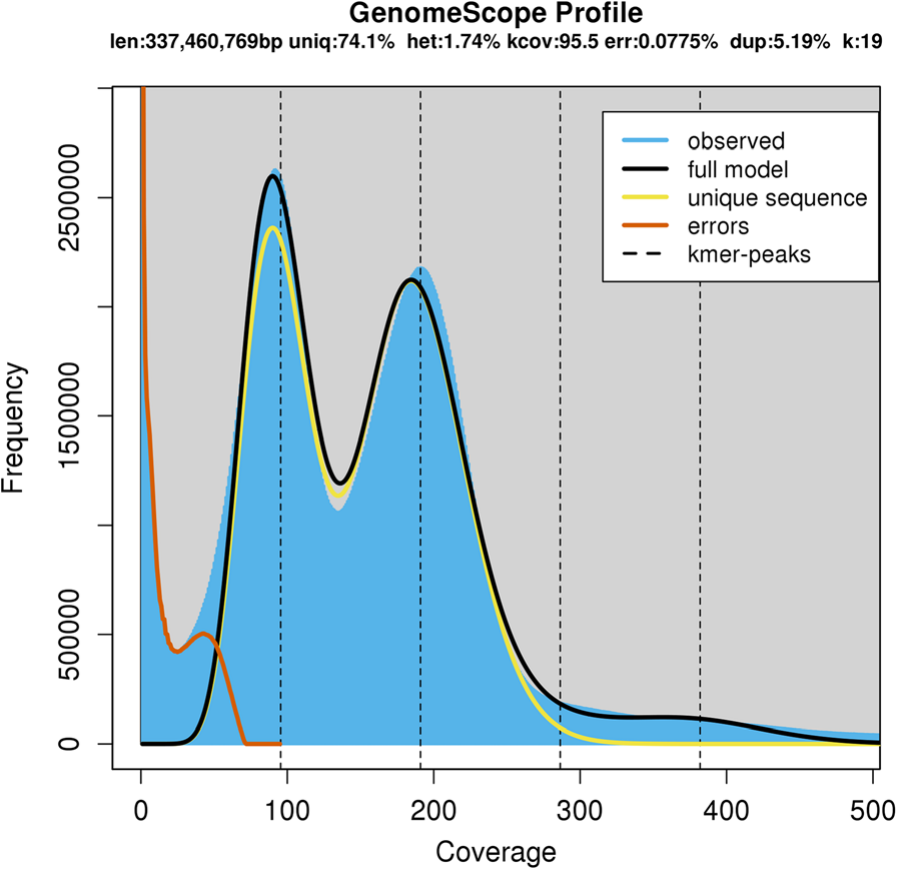
GenomeScope result of ‘Sultanina’ genome. The first peak located at coverage 92X corresponds to the heterozygous peak. The second peak at coverage 191X, corresponds to the homozygous peak. Estimate of the heterozygous portion is 1.74%

**Fig. 2:**
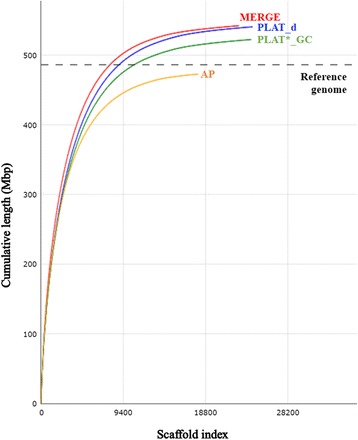
Cumulative assembly length. Scaffold lengths for all four assemblies (AP = orange, PLAT_d = blue, MERGE = red, and PLAT*_GC = green). The scaffolds are ordered from the largest to smallest (x-axis) and scaffold cumulative size is on y-axis. The horizontal dotted line represents *V. vinifera* reference genome (PN40024 12X.v2)

A 1.74% heterozygosity was estimated for the ‘Sultanina’ genome (Fig. 1). The PLAT_d had a greater number of scaffolds >10 kbp and greater estimated useful portion of scaffold sequences (> 25 kbp) than AP (Table 1, Additional file 1 a). The PLAT_d assembly cumulative size (Quast tool) indicated the median and cumulative scaffold size was greater than AP (Fig. 2, Additional file 1).

**Table 1:** Assemblathon statistics for the four ‘Sultanina’ assemblies

Details	AP	PLAT_d	MERGE	PLAT*_GC
Number of scaffolds	17,920	24,112	22,566	23,981
Total size of scaffolds	472,715,607	540,637,988	542,245,619	522,430,188
Number of scaffolds > 1 kbp	17,918	24,099	22,554	23,962
Number of scaffolds > 10 kbp	8129	9512	8897	9251
Number of scaffolds > 100 kbp	1101	1214	1302	1132
Scaffold %N	10.7	6.6	6.4	1.2
N50 scaffold length	78,751	72,490	82,589	71,610
NG50 scaffold length	75,014	83,858	95,278	78,173
Number of contigs	68,261	75,914	72,375	32,394
Number of contigs in scaffolds	62,855	65,362	62,706	14,965
Read map back, 0 mismatch using1.022 billion reads (Gr)	0.889 Gr	0.961 Gr	0.964 Gr	0.97 Gr
	86.7%	94.0%	94.3%	94.9%

The four assemblies (AP [12], PLAT_d, MERGE, and PLAT*_GC) were evaluated using Assemblathon script with scaffold size limited to 1 kbp. Complete assemblathon results are presented in Additional file 1

Comparison of the gap closing assemblies indicated that the PLAT*_GC assembly had fewer and larger contigs than all other assemblies (Additional file 1 a, c). MERGE had the largest number of scaffolds > 100 kbp and provided a greater mean and median scaffold size (Table 1, Additional file 1). The cumulative assembly analysis by QUAST tool indicated that the median and cumulative scaffold size and useful portion of scaffolds (> 25 kbp) was greater for MERGE and PLAT*_GC than AP.

A zero-mismatch map back of the filtered reads showed that PLAT_d, MERGE, and PLAT_GC (bold) was >94%, thus maintaining greater identity to the original sequence data in contrast to AP with 86.7% read map back.

Alignments of assemblies with the *V. vinifera* reference genome and SNP identification.

**Fig. 3:**
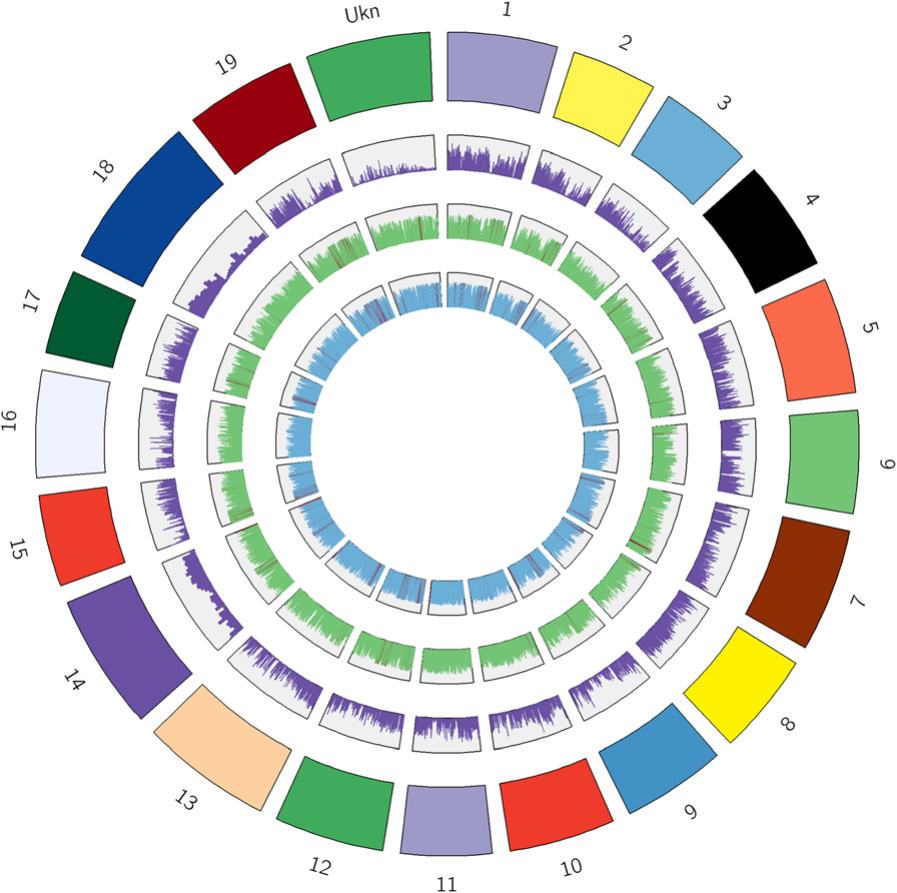
Top hits length of AP and PLAT*_GC Sultanina assemblies with *V. vinifera* reference genome (PN40024 12X.v2). Outer ring = chromosomes of reference genome *V. vinifera* (PN40024 12X.v2); Second ring = SNP calls histogram (purple) for each chromosome (variants by chromosome relative to the *V. vinifera* (PN40024 12X.v2) reference genome in 100 kbp bins). The 2 inner most rings represent 95% identity of 1 kbp alignment length for AP assembly (third ring, green), and PLAT*_GC (fourth ring, blue). The red bars in the third and fourth rings represent 100% identity and minimum 1000 bp alignment with *V. vinifera* reference genome (details are in Table 2)

Additional high quality SNPs (1,205,953) were identified in the analysis with the recently released *V. vinifera* reference genome (PN40024 12X.v2) adding to the previously published (1,197,594) for Sultanina [12]; the variant distribution by chromosome is shown in Fig. 3. A greater number of the scaffolds from the PLATANUS derived assemblies than the AP assembly aligned to the *V. vinifera* reference genome (PN40024 12X.v2) with 67–69% of scaffolds showing an alignment of at least minimum 95% identity and 1000 bp alignment length [18] (Table 2). PLAT_d had the greatest number (615) of scaffolds with 100% identity (Table 2). A comparison of the final gap-close assembly PLAT*_GC and AP at 100% and > 1000 bp alignment showed that the PLATANUS assemblies provided greater synteny with the inbred *V. vinifera* (PN40024 12X.v2) reference genome than with ALLPATH-LG assembly (AP) (Fig. 3). For greater clarity only the AP and PLAT*_GC alignments are shown in Fig. 3.

**Table 2:** Statistics of alignment of four Sultanina de novo assemblies with *V. vinifera* (PN40024 12X.v2) reference genome

	AP	PLAT_d	MERGE	PLAT*_GC
Total Scaffold number > 1 kbp	17,920	24,112	22,566	23,981
Top-hits-length (95% identity and minimum 1kbp alignment length^a^	99,297,844	131,213,373	126,718,587	152,634,079
Number of scaffolds aligned (95% identity and minimum 1 kbp alignment, % total scaffolds)	10,892 (61%)	16,462 (68%)	15,193 (67%)	16,572 (69%)
Top-hits-length (100% identity and minimum 1 kbp alignment length	470,236	1,824,500	1,604,407	1,509,723
Number of scaffolds aligned, (100% identity and minimum 1 kbp alignment)	187	615	560	558
SyMAP synteny blocks (anchors having alignment lengths >10 kb)^b^	12,112	13,448	13,421	13,412

^a^One-to-one relationship between scaffolds for each assembly and the reference genome were constructed according to the longest alignment for each scaffold, and the total of the alignment lengths (top-hits-length) were calculated

^b^Calculated with SyMAP [42] used to map with the *V. vinifera* reference genome

**Fig. 4:**
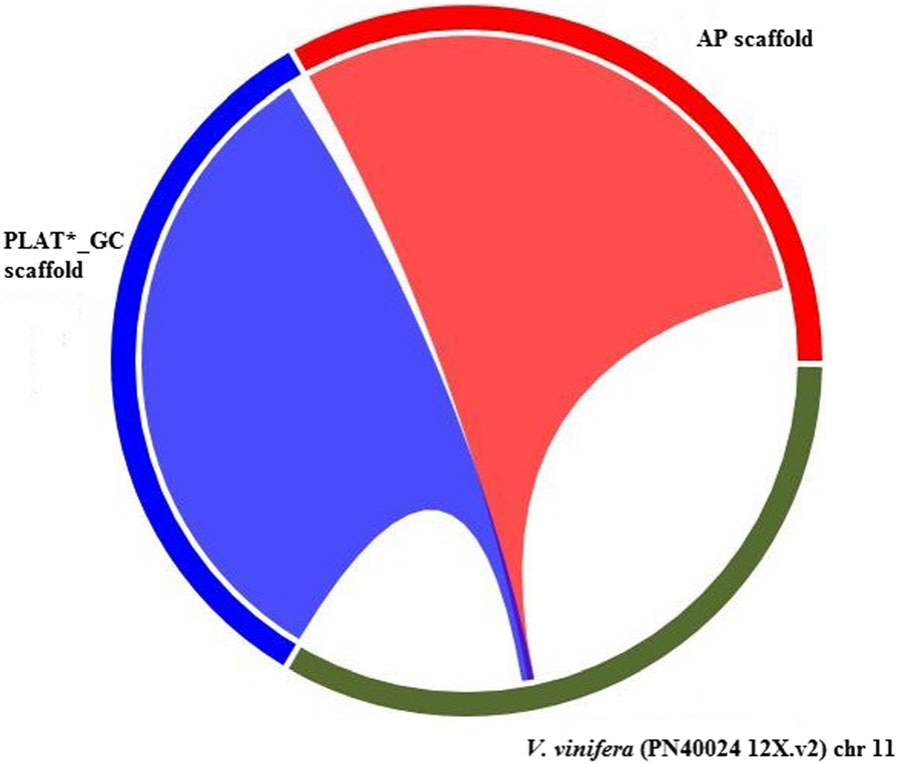
Largest AP and PLAT*_GC scaffold aligned with a region of chromosome 11 for reference genome *V. vinifera* (PN40024 12X.v2). The AP and PLAT*_GC seedless grapevine scaffolds are shown in an enlargement of position 6,840,000 to 7,190,000 of *V. vinifera,* (PN40024 12X.v2) chromosome 11. The *V. vinifera,* (PN40024 12X.v2) chromosome 11 is green, AP scaffold is red (241 kbp with 3,756,111 zero-mismatch reads mapped back) and PLAT*_GC is blue (315 kbp with 8,845,593 zero-mismatch reads mapped back)

An example of scaffold long range fidelity to original sequence reads is illustrated in Fig. 4. Comparison of the AP and PLAT*_GC longest scaffold aligning to *V. vinifera* (PN40024 12X.v2) chromosome 11 and the zero-mismatch read map back showed a greater incorporation of original reads per kbp of scaffold in the PLAT*_GC scaffold than in the AP scaffold (Fig. 4).

### Synteny analysis

**Fig. 5:**
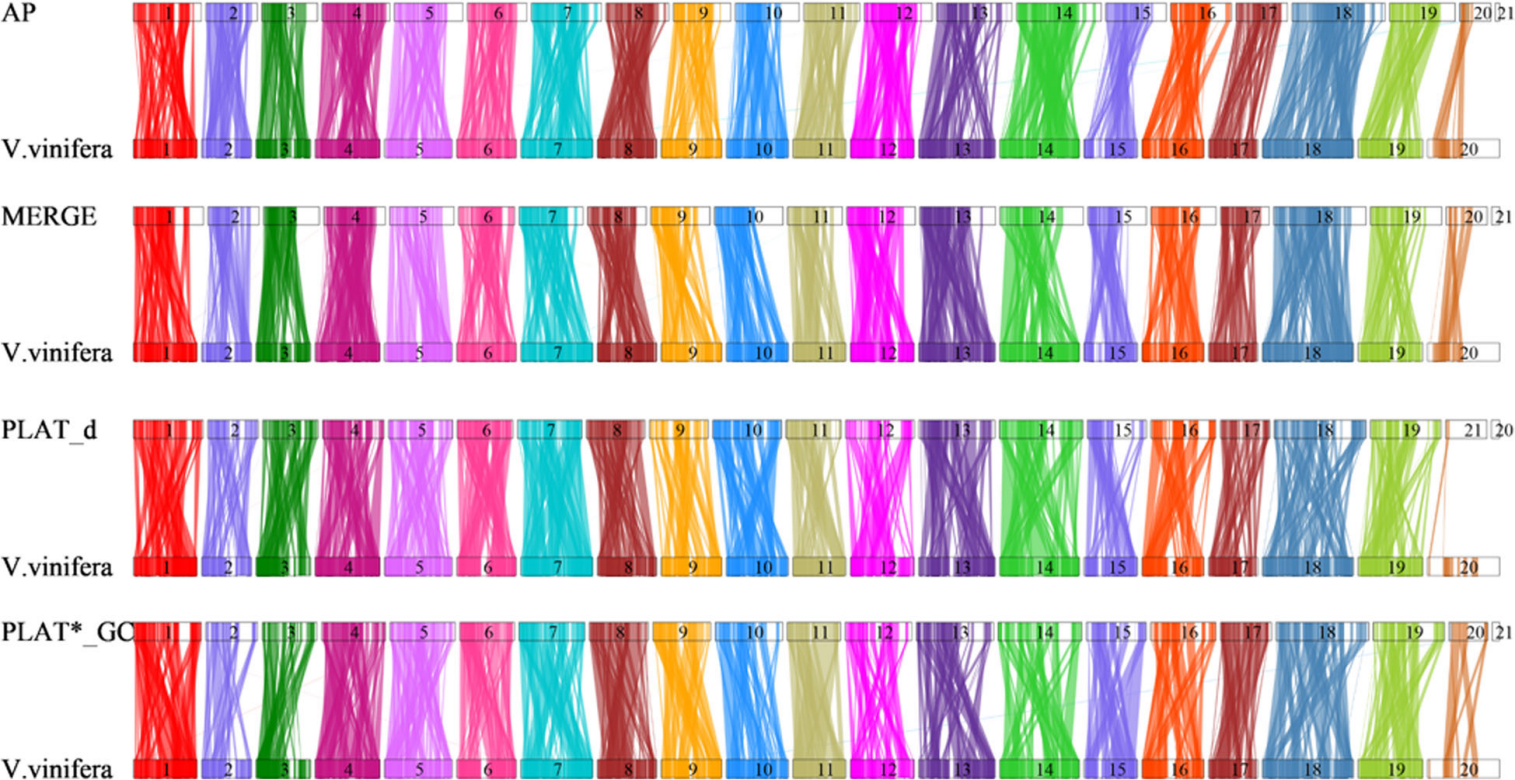
Synteny analysis of four ‘Sultanina’ assemblies with the *V. vinifera* (PN40024 12X.v2) reference genome

The number of extended conserved synteny blocks (> 10 kb) in comparison with the inbred *V. vinifera* (PN40024 12X.v2) were greater for the three de novo assemblies and PLAT_d, MERGE, and PLAT*_GC had ≥ 2300 more synteny blocks than the AP. SyMAP [42] visualization of the synteny blocks show that the MERGE synteny blocks map similarly as the AP blocks do with the inbred *V. vinifera* reference genome, although there are differences particularly for chromosomes 1, 3, 14, 16 and 18 (Fig. 5). The synteny blocks in PLAT_d and PLAT*_GC are very similar across all chromosomes (Fig. 5). Synteny differences appear with all assemblies and the inbred *V. vinifera* reference genome and may indicate real rearrangements or assembly difficulties with repeat regions, short reads and the heterozygosity of the Sultanina.

### Assembly validation by BAC and EST sequences of *V. vinifera* sequences and BUSCO proteins

The four assemblies were compared using *V. vinifera* BAC [19] and *V. vinifera* EST sequences from NCBI. Alignments showed that more BACs aligned to the PLATANUS related assemblies (PLAT_d, MERGE and PLAT*_GC) than to the AP assembly. A similar number of the EST sequences were aligned to all four assemblies using GMAP [29] with a greater number mapped to PLAT*_GC when the more stringent parameters (min-identity 90% and min-coverage 70%) were used (Table 3).

**Table 3:** BAC and EST validation information for all four assemblies

Details		AP	PLAT_d	MERGE	PLAT*_GC
BAC sequences: 120,148 BAC bp 66,371,065	Top-hits-length (bp)	55,484,200	59,330,132	59,387,356	59,520,643
	Number of BAC sequences	108,172	111,600	111,649	112,325
	Top-hits-length with 90% identity and 90% coverage (bp)	46,565,227	53,082,062	52,959,509	52,980,593
	Number of BAC sequences	83,879	95,375	95,147	95,152
EST sequences: 511,685 EST bp: 320,415,647	Top-hits-length (bp)	264,199,117	265,484,201	267,151,205	268,356,483
	Number of EST sequences	463,419	466,569	467,024	467,548
	Top-hits-length with 90% identity and 70% coverage (bp)	237,629,769	237,868,274	241,453,791	244,324,283
	Number of EST sequences	393,826	395,439	400,650	405,329

One-to-one relations between each BAC, EST and scaffolds were constructed according to the longest alignment for each BAC and EST and the total of those alignment lengths (top-hits-length) was calculated

The de novo gene prediction using seedless RNA-seq data predicted 26,515 coding sequences for the AP assembly and greater than 30,000 were predicted in the three new PLATANUS derived assemblies (Table 4). These de novo predictions resulted in a greater number of hits with the latest *V. vinifera* (PN40024 12X.v2) VCOST.v3 proteins [14] (Table 4). Comparison of the predicted proteins with *V. vinifera* (PN40024 12X.v2, VCOST.v3) proteins showed 13,571 in common across all assemblies with 30,544 in PLAT*_GC in common with VCOST.v3 proteins (Table 4, Additional file 2: Figure S1). More predicted proteins of PLAT*_GC were in common with the *V. vinifera* (PN40024 12X.v2, VCOST.v3) proteins than with any of the other assemblies (Table 4, Additional file 2: Figure S1).

**Table 4:** Predicted genes for seedless assemblies and number of *V. vinifera* (PN40024 12X.v2, VCOST.v3) specific BLASTX hits

	AP	PLAT_d	MERGE	PLAT*_GC
Total Number of coding sequences	26,515	30,433	30,346	30,544
#BLASTX Hits with *V. vinifera* (VCOST.v3) only	26,411	30,340	30,242	30,434
# with no hit to *V. vinifera* (VCOST.v3)	104	93	104	110
#BLASTX Hits of no hit *V. vinifera* (VCOST.v3) to NCBI	12	5	14	8
# with no BLASTX hit in NCBI	92	88	90	102

All previously identified “novel” seedless genes [12] were found in the VCOST.v3 gene models. A greater number of PLAT*_GC non-hits (against *V. vinifera* PN40024 12X.v2), were also identified by BLASTP for PLAT*GC than AP (Additional file 3 a). The enzyme classifications were similar for AP and PLAT*_GC. A greater number of GO functional categories were identified in PLAT*_GC than AP (Additional file 3 c); however those characterized by InterPro and KEGG pathways were similar in all assemblies (Additional file 3 d,e).

BUSCO analysis showed that all four assemblies had similar numbers of conserved proteins. The short read assemblies varied from 92 to 94% of total BUSCO proteins searched, with AP and PLAT*_ GC both containing 94% of the BUSCO proteins (Table 5).

**Table 5:** BUSCO validation for all four assemblies

BUSCO details	AP	PLAT_d	MERGE	PLAT*_GC
Complete BUSCOs (C)	1356	1333	1344	1351
Complete and single-copy BUSCOs (S)	1320	1308	1316	1319
Complete and duplicated BUSCOs (D)	36	25	28	32
Fragmented BUSCOs (F)	33	38	33	34
Missing BUSCOs (M)	51	69	63	55
Total BUSCO groups searched	1440	1440	1440	1440

### Plant transcription factor analysis

**Fig. 6:**
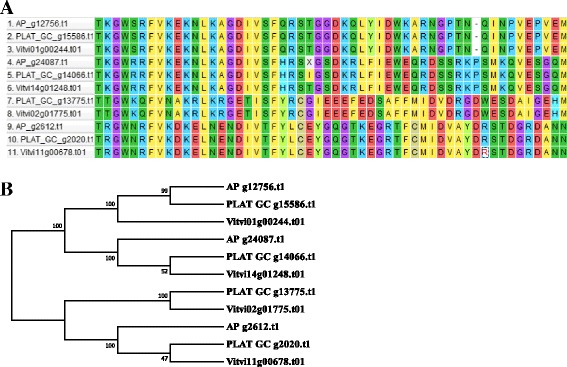
RAV transcription factor sequence alignment. **a** RAV family protein alignment for *V. vinifera* (PN40024 12X.v2), AP and PLAT*_GC. **b** RAV family phylogenetic tree (1000 permutations)

The plant transcription factors (TFs) were identified in all four assemblies and the latest (2017) *V. vinifera* (PN40024, VCOST.v3) gene models (Additional file 4). A total of 1334 TFs were predicted for AP in 58 different TF families. There were 1399 and 1388 TFs predicted for PLAT_d and MERGE, respectively. The ‘GRF’ TF was not found in PLAT_d and MERGE and ‘STAT’ TF was not predicted in PLAT_d. PLAT*_GC predicted total 1433 TFs and covered all the 58 TFs families found in *V. vinifera* VCOST.v3 (Additional file 4). In general, there were fewer ERF and MYB TFs identified in the de novo seedless grape assemblies; however, there were more MYB_related TFs identified in PLAT*_GC, pointing to potential problems in assembling these TF families with short reads. An example comparison of the RAV TFs from the AP2/ERF super family is shown in Fig. 6. The RAV TF gene family, is important in growth and development and response to stress, contains a B3 domain in addition to one AP2/ERF domain. There are four genes identified in *V. vinifera* (PN40024, VCOST.v3) and PLAT*_GC and only three were predicted in AP (Fig. 6), The phylogenetic tree indicated that the PLAT*_GC are more similar to the *V. vinifera (*PN40024*)* genes.

## Discussion

The decreased cost and speed of next generation sequencing provides the opportunity for new sequencing and exploration of genome variation in important crop cultivars, thus allowing identification of novel genes and polymorphisms that may be useful in marker assisted selection. However, assembly of heterozygous species like grapevine is a difficult task and is addressed by only a few assembly algorithms [8, 11]. Previously, *V. vinifera* ‘Sultanina’ genome was assembled with the ALLPATHS-LG assembler in default mode using the ‘HAPLOIDIFY’ option to minimize heterozygosity issues [12]. However, maintaining the heterozygosity in the de novo genome assembly for use in identifying structural and allele variants is a very important consideration [6, 8–10]. Recently, some diploid heterozygous plant species have been assembled using solely Illumina short reads and the PLATANUS assembler [43–45]. In addition to the published Sultanina grapevine AP assembly [12], a lotus (*Nelumbo nucifera* Gaertn.) genome was assembled using ALLPATH-LG [46]. The high quality of the lotus genome assembly was attributed to a high degree of homozygosity observed in the sequenced lotus variety [46]. The 0.03% lotus heterozygosity is lower than has been achieved by inbreeding other crops [46] and much lower than 1.74% heterozygosity estimated in this study for the Sultanina sequence.

The PLATANUS assembler [11] was developed to address the challenge of de novo assembling of heterozygous genomes and is beginning to be used for a diversity of plant and animal species [43]. Other challenges in de novo plant genome assembly include a highly fragmented assembly that is less suitable for use in other studies, such as comparative genomics and correlations with linkage maps due to genome complexity. The capability of merging assemblies from different algorithms [15] and parameters [11] also provides the opportunity to develop improved assemblies with greater contiguity [15]. Therefore, in this study different assembly algorithms, merging the different assemblies and transcriptomic data were used to develop improved assemblies and gene predictions. PLATANUS software [11] was used in default mode to provide a baseline assembly to compare with ALLPATHS-LG assembly (AP) [12]. Development of less fragmented and increased scaffold sized assemblies was approached through two separate strategies: 1) An iterative merging strategy using METASSEMBLER to combine two assemblies from different assemblers (MERGE); 2) A PLAT*_GC assembly was developed by altering branch cutting and contig scaffolding steps. In the PLAT*_GC assembly, merging more heterozygous contigs in the ‘assemble’ step was achieved by changing ‘-u’ and ‘-d’ parameters respectively. After the assembler and scaffold step the scaffold still contained 6.7 %N and this was reduced to 1.2% using GapCloser [16] (Additional file 1 b). The major impact of GapCloser [16] was in improving the contig statistics with an increase in large contigs and a greater amount of useful scaffold length (>25 kbp) with greater continuity as evidenced by the lower %Ns in the PLAT*_GC scaffolds. These results suggest that we were able to merge small contigs into larger contigs to a greater extent than was obtained using ALLPATHS-LG or METASSEMBLER. A test of processing time indicated that ALLPATHS-LG software in default mode required over 3 days to assemble whereas PLATANUS in default was slightly more efficient at 2.5 days for the same assembly. It should be noted that although PLATANUS [11] has low memory requirements (800 Gb) for operating in default mode, the parameter modifications require greater memory availability (900 Gb) for the assembling process to run to completion.

The new assemblies each had distinct characteristics. For example, the AP assembly had a greater median scaffold length but otherwise had lower sequence coverage and greater gaps (scaffold %N) than the PLAT_d, MERGE and PLAT*_GC assemblies. Comparison of the three de novo assemblies developed here indicated that the iterative merging resulted in greater scaffold size and an overall increase in assembly size for MERGE assembly; however, a similar gap level (6% N) was found for MERGE and PLAT_d. In contrast, PLAT*_GC had greater contig size prior to scaffolding and lower gaps (1.2% N) in final assembly than AP, PLAT_d and MERGE. There was an increased fidelity to the heterozygosity of the original read sequences in the PLAT*_GC assembly than found in AP as evidenced by the zero-mismatch map back to the longest scaffolds in AP and PLAT*_GC. It is important to note that the haploidify option in ALLPATHS-LG statistically selects one branch and discards the other; therefore, reducing polymorphic regions and the assembly heterozygosity, which can result in loss of information useful for SNP calling and marker development. Thus the observed reduction in read map back for AP is most likely a result of the haploidify processes which provides a greater consensus sequence. The AP assembly did not capture all members of the RAV gene family while all four were found in the PLAT*_GC assembly and they are more similar to the inbred *V. vinifera* (PN40024 12X.v2 VCOST.v3) RAV family members.

Comparative analysis of different assembling strategies provides the opportunity to evaluate and improve genome assembly quality, thus improving the potential to predict novel genes and identifying informative SNPs for marker selection. Genotyping by sequencing provides the ability to generate high-resolution genetic maps at a low cost; however, for highly heterozygous species like grapevine, missing data and heterozygote under calling make it more difficult to create dense genotype by sequencing genetic maps [6]. In this study, we obtained a greater conformity with the sequence reads and thus a greater potential maintenance of heterozygosity using PLATANUS software [11].

Further functionality of an assembly can be addressed by comparing gene models. In contrast to the previous AP publication [12] de novo predicted coding sequences were generated for all four assemblies using RNA-seq data of seedless grape from NCBI [13]. The gene prediction for the PLAT*_GC presented here identified 4000 more transcripts than in AP assembly. In addition, the previously predicted “novel” genes [12] were found in the latest *V. vinifera* reference genome VCOST.v3 annotation [14]. The PLAT*_GC improved assembly with its greater accuracy also provides the opportunity to use it for developing hybrid assemblies with long read technology as has been done for citrus and other woody plants [47–49].

## Conclusions

This study provides greater resources for transcriptomic analyses, more informative SNP calling for genotype by sequencing data, and an improved assembly for genetic research in the seedless grapevine. The PLATANUS and METASSEMBLER software allowed development of larger assemblies with larger and more contiguous scaffolds. After comparative analysis of all four assemblies we conclude that PLAT*_GC assembly provides greater fidelity to the original sequences and greater continuity within scaffolds. To enable further research studies a pseudo_chromosomal_assembly of PLAT*_GC assembly with gene prediction and annotation have been provided (http://openprairie.sdstate.edu/vitis_vinifera_sultanina/1). The greater fidelity to the original sequence reads maintained in the PLAT*_GC assembly makes it very useful for future use with long reads for a hybrid assembly or other genetic, mapping and breeding-related applications.

## Additional files

Additional file 1: Assembly statistics for four *Vitis vinifera* ‘Sultanina’ de novo assemblies. a: Assembly statistics for four *Vitis vinifera* ‘Sultanina’ de novo assemblies. All assemblies evaluated using Assemblathon metrics and scaffold size limited to 1 kbp. b: Assembly statistics for PLAT*_GC assembly steps. All assemblies were evaluated using Assemblathon metrics and scaffold size limited to 1kbp. c: Assembly statistics for four *Vitis vinifera* ‘Sultanina’ de novo assemblies. All full assemblies (scaffold ≥ 500 nt) were evaluated using Assemblathon metrics. (XLSX 21 kb)

Additional file 2: Figure S1. Protein alignment with *V. vinifera* (PN40024 12X.v2, VCOSTv.3 proteins. a. Orthologous proteins for all seedless grape assemblies in relation to the *V. vinifera* VCOST.v3 (*V. vinifera* V3). b. Comparison of AP with the three de novo seedless assmemblies. (JPEG 107 kb)

Additional file 3: Functional characterization of predicted genes for the four assemblies using Blast2GO, BLASTX and BLASTP. (XLSX 11 kb)

Additional file 4: Plant transcription factor identification for all four assemblies and *V. vinifera* (VCOST.v3) using PlantTFDB. (XLSX 15 kb)

## Abbreviations

AP: ALLPATHS-LG assembly
Gr: Giga reads (billion reads)
MERGE: Default PLATANUS assembly merged with ALLPATHS-LG assembly
PLAT*_GC: PLATANUS assembly with parameter modifications and Gap Closer
PLAT_d: Default PLATANUS assembly
SNP: Single nucleotide polymorphism
