# Supplementary figures and images for: Comparison of three assembly strategies for a heterozygous seedless grapevine genome assembly
Source: BMC Genomics. 2018 Jan 17;19:57. doi: 10.1186/s12864-018-4434-2 (PMC5773036; doi:10.1186/s12864-018-4434-2)

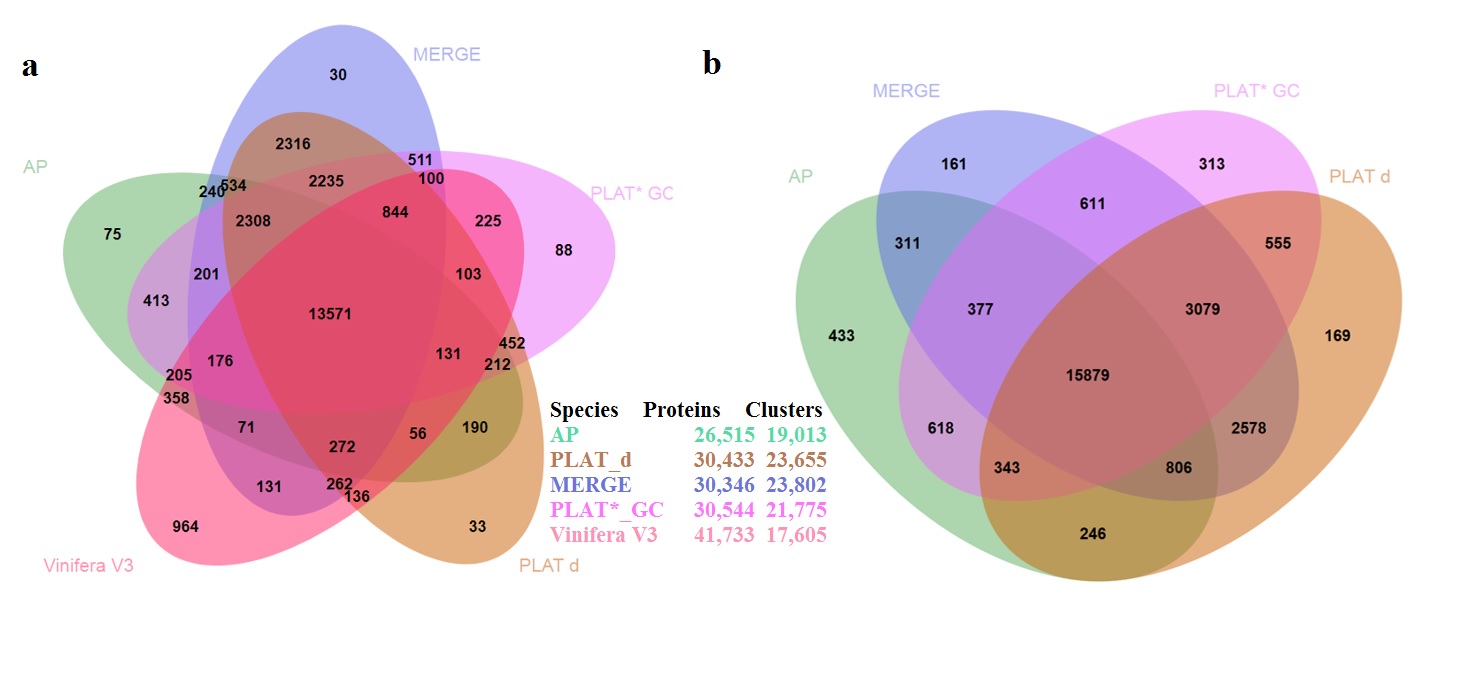

Supplement: Supplementary file 2 — Protein alignment with V. vinifera (PN40024 12X.v2, VCOSTv.3 proteins. a. Orthologous proteins for all seedless grape assemblies in relation to the V. vinifera VCOST.v3 (V. vinifera V3). b. Comparison of AP with the three de novo seedless assmemblies. (JPEG 107 kb) [file 12864_2018_4434_MOESM2_ESM.jpg]
